# Autonomic Neuroscience: articles of interest appearing in other Frontiers journals

**DOI:** 10.3389/fnins.2012.00184

**Published:** 2012-12-21

**Authors:** Vaughan G. Macefield, Joel C. Bornstein

**Affiliations:** ^1^School of Medicine, University of Western SydneySydney, NSW, Australia; ^2^Department of Physiology, The University of MelbourneMelbourne, VIC, Australia

Dear Readers of Frontiers in Autonomic Neuroscience,

As you are all aware, autonomic neuroscience is a multidisciplinary field, in which authors choose a journal based on its perceived impact but, more importantly, on its relevance and readership. Together, the Frontiers journals are broad in scope, yet the areas covered by individual journals within the Frontiers stable are necessarily narrower. Now, to facilitate your use of the Frontiers articles we are going to highlight those articles published in our sister journals that are relevant to us working in autonomic neuroscience.

So, here is a wrap-up of articles you may have missed over the last few months.

## Frontiers in integrative physiology

Published on 1 Oct 2012

**Autonomic markers of emotional processing: skin sympathetic nerve activity in humans during exposure to emotionally charged images**

Rachael Brown, Cheree James, Luke A. Henderson and Vaughan G. Macefield

Published on 07 Aug 2012

**Arterial baroreflex control of muscle sympathetic nerve activity under orthostatic stress in humans**

Masashi Ichinose and Takeshi Nishiyasu

Published on 24 Jul 2012

**Sympathetic regulation of vascular function in health and disease**

Rosa M. Bruno, Lorenzo Ghiadoni, Gino Seravalle, Raffaella Dell'Oro, Stefano Taddei and Guido Grassi

Published on 18 Jul 2012

**Microneurographic research in women**

Qi Fu

Published on 17 Jul 2012

**Upper gastrointestinal dysmotility after spinal cord injury: is diminished vagal sensory processing one culprit?**

Gregory M. Holmes

Published on 09 Jul 2012

**Plasticity of TRPV1-expressing sensory neurons mediating autonomic dysreflexia following spinal cord injury**

Leanne M. Ramer, A. Peter van Stolk, Jessica A. Inskip, Matt S. Ramer and Andrei V. Krassioukov

Published on 26 Jun 2012

**Autonomic dysfunction in heart failure and renal disease**

Jacqueline K. Phillips

Published on 25 Jun 2012

**Somatosympathetic vasoconstrictor reflexes in human spinal cord injury: responses to innocuous and noxious sensory stimulation below lesion**

Vaughan G. Macefield, Alexander R. Burton and Rachael Brown

Published on 07 Jun 2012

**Association of cardiac baroreflex sensitivity with blood pressure transients: influence of sex and menopausal status**

Jill N. Barnes, Luke J. Matzek, Nisha Charkoudian, Michael J. Joyner, Timothy B. Curry and Emma C. Hart

Published on 23 May 2012

**Firing patterns of muscle vasoconstrictor neurones in respiratory disease**

Vaughan G. Macefield

Published on 03 May 2012

**Advantage of recording single-unit muscle sympathetic nerve activity in heart failure**

Hisayoshi Murai, Masayuki Takamura and Shuichi Kaneko

Published on 26 Apr 2012

**Sympathetic responses to central hypovolemia: new insights from microneurographic recordings**

Kathy L. Ryan, Caroline A. Rickards, Carmen Hinojosa-Laborde, William H. Cooke and Victor A. Convertino

Published on 10 Apr 2012

**Autonomic regulation during quiet and active sleep states in very preterm neonates**

Sina Reulecke, Steffen Schulz and Andreas Voss

Published on 20 Feb 2012

**Evidence and consequences of the central role of the kidneys in the pathophysiology of sympathetic hyperactivity**

Eva E. Vink and Peter J. Blankestijn

Published on 07 Feb 2012

**Advances in sympathetic nerve recording in humans**

Elisabeth Lambert, Dagmara Hering, Markus Schlaich and Gavin Lambert

Published on 02 Feb 2012

**Effects of renal denervation on sympathetic activation, blood pressure, and glucose metabolism in patients with resistant hypertension**

Markus P. Schlaich, Dagmara Hering, Paul Sobotka, Henry Krum, Gavin W. Lambert, Elisabeth Lambert and Murray D. Esler

Published on 12 Jan 2012

**Prognostic indicators of cardiovascular risk in renal disease**

Cara M. Hildreth

Published on 03 Jan 2012

**Brain renin–angiotensin system in hypertension, cardiac hypertrophy, and heart failure**

Luciana A. Campos, Michael Bader and Ovidiu C. Baltatu

## Frontiers in cardiac electrophysiology

Published on 24 Sep 2012

**Cyclical modulation of human ventricular repolarization by respiration**

Ben Hanson, Jaswinder Gill, David Western, Michael P. Gilbey, Julian Bostock, Mark R. Boyett, Henggui Zhang, Ruben Coronel and Peter Taggart

## Frontiers in computational physiology and medicine

Published on 21 Sep 2012

**Time scales of autonomic information flow in near-term fetal sheep**

M. G. Frasch, B. Frank, M. Last and T. Müller

## Frontiers in exercise physiology

Published on 23 Oct 2012

**Sympathetic limitation of exercise hyperemia: even hypoperfused muscle is not exempted**

D. J. Duncker and I. H. Heinonen

## Frontiers in clinical and translational physiology

Published on 7 Sep 2012

**Cardiovascular autonomic nervous system function and aerobic capacity in type 1 diabetes**

Harriet Hägglund, Arja Uusitalo, Juha E. Peltonen, Anne S. Koponen, Jyrki Aho, Suvi Tiinanen, Tapio Seppänen, Mikko Tulppo and Heikki O. Tikkanen
